# Comparison between newly diagnosed hypertension in diabetes and newly diagnosed diabetes in hypertension

**DOI:** 10.1186/s13098-019-0465-3

**Published:** 2019-08-23

**Authors:** Chang-Yuan Liu, Wei Zhang, Li-Nong Ji, Ji-Guang Wang, for the ATTEND investigators

**Affiliations:** 1 Centre for Epidemiological Studies and Clinical Trials, Shanghai Key Laboratory of Hypertension, The Shanghai Institute of Hypertension, Department of Hypertension, Ruijin Hospital, Shanghai Jiaotong University School of Medicine, Ruijin 2nd Road 197, Shanghai, 200025 China; 2 Department of Endocrinology, Renmin Hospital, Peking University, Beijing, China

**Keywords:** Hypertension, Diabetes mellitus, Body mass index, Heart rate, Albuminuria, Left ventricular hypertrophy

## Abstract

**Background:**

Hypertension and diabetes mellitus are often jointly present, especially in early onset cases of either disease. We investigated clinical characteristics of hypertensive patients with newly diagnosed diabetes and diabetic patients with newly diagnosed hypertension.

**Methods:**

Our study subjects were recruited in a China nationwide multicenter registry of hypertension and diabetes (n = 2510). We performed logistic regression to compare patients seen for hypertension in cardiology, with newly diagnosed diabetes (n = 137) and patients seen for diabetes mellitus in endocrinology, with newly diagnosed hypertension (n = 155). Albuminuria was defined as a urinary albumin-to-creatinine ratio of ≥ 30 mg/g, and left ventricular hypertrophy according to the Cornell product index.

**Results:**

These two groups of patients with both hypertension and diabetes mellitus were similar in most of the characteristics (*P *≥ 0.06). However, hypertensive patients with newly diagnosed diabetes, compared to diabetic patients with newly diagnosed hypertension, had a significantly greater body mass index (26.3 vs. 25.4 kg/m^2^, *P *= 0.03) and slower heart rate (73.7 vs. 78.1 beats/min, *P *= 0.01). In logistic regression analyses adjusted for sex (48.3% women) and age (mean 60.0 ± 11.5 years), the odds ratio for newly diagnosed diabetes mellitus versus newly diagnosed hypertension was 1.27 (95% CI 1.03–1.56) and 0.80 (95% CI 0.66–0.96) for body mass index (+ 3 kg/m^2^) and heart rate (+ 10 beat/min), respectively. Hypertensive patients with newly diagnosed diabetes also had a lower prevalence of albuminuria (16.0% vs. 30.1%, *P *= 0.02) and slightly and non-significantly higher prevalence of left ventricular hypertrophy (5.1% vs. 1.9%, *P *= 0.14) than diabetic patients with newly diagnosed hypertension.

**Conclusions:**

Earlier or later onset of hypertension than diabetes mellitus may have different risk factors and organ damage.

## Background

Hypertension and diabetes mellitus are closely related [1]. These two disorders are often jointly present, and the joint presence of the two disorders confers high risks of target organ damage [2, 3] and cardiovascular morbidity and mortality [4]. In a recent China nationwide registry, we found that the prevalence of both hypertension and diabetes mellitus was disproportionate between hypertensive patients seen in cardiology and diabetic patients seen in endocrinology, being 32.9% and 58.9%, respectively [5]. Because the prevalence of hypertension in the general population was higher than that of diabetes mellitus, one possible explanation could be that only a fraction of hypertension is related to diabetes mellitus, for instance, the so-called obesity-associated hypertension. Nonetheless, other reasons may also play a part. These two disorders may share similar pathogenic pathways or one causes another. Diabetes mellitus increases arterial stiffness and in turn systolic pressure and pulse pressure. In addition, the use of some antihypertensive drugs, such as the combination of high-dose thiazides and β-blockers, [6] is associated with an increased risk of diabetes mellitus. Some antidiabetic drugs may induce sodium and fluid retention, [7] which may also cause a rise in blood pressure. It is therefore intriguing to investigate which group of hypertensive patients would develop diabetes mellitus and which group of diabetic patients would develop hypertension.

Our China nationwide registry in the settings of cardiology and endocrinology offers a unique opportunity to investigate the newly diagnosed hypertension in diabetes and the newly diagnosed diabetes mellitus in hypertension [5]. In the present analysis, we compared the clinical characteristics between newly diagnosed hypertension in diabetes mellitus and newly diagnosed diabetes mellitus in hypertension.

## Methods

### Study participants

Our study participants were recruited from the abovementioned China multicentre registry, which was a cross-sectional study carried out in the departments of cardiovascular and endocrine medicine of hospitals from June 2011 to March 2012 [5]. The study protocol was described in detail previously [5]. In brief, we registered consecutive patients with previously diagnosed hypertension from the departments of cardiovascular medicine and those with previously diagnosed diabetes mellitus from the departments of endocrine medicine. The ethics committees of all participating hospitals approved the study protocol. All subjects gave written informed consent.

To be eligible for inclusion, a patient had to be at least 20 years old, and was able to attend two clinic visits 2 to 5 days apart. At the first clinic visit, physicians administered a standardized questionnaire to collect information on medical history, lifestyle, and use of medications. Blood pressure and anthropometry were measured. At the second clinic visit, blood pressure was measured for the second time. Venous blood samples were drawn after overnight fasting for measurements of plasma glucose, glycosylated haemoglobin A1 (HbA1c) and serum lipids. Morning void urine samples were collected for urinary measurements. Oral glucose tolerance test (OGTT) was performed in the hypertensive patients without known diabetes mellitus [5]. We excluded pregnant women, patients with a history of type 1 diabetes mellitus, or patients participating in another study in the past 3 months.

A total of 2510 patients were registered from 20 departments of cardiology (n = 1330) and 20 departments of endocrinology (n = 1180) of 25 hospitals [5]. The present analysis, however, was restricted to 137 hypertensive patients seen in cardiology with newly diagnosed diabetes mellitus by an oral glucose tolerance test (OGTT) and to 155 diabetic patients seen in endocrinology with newly diagnosed hypertension according to blood pressure on two clinic visits.

### Clinic blood pressure and OGTT

Blood pressure was measured using the validated Omron HEM-7201 automated oscillometric blood pressure monitor (Omron Healthcare, Kyoto, Japan) at the first and second clinic visits. On each of the two occasions, three consecutive blood pressure readings were obtained in the seated position after the subjects had rested for at least 5 min. These six readings on two clinic visits were averaged for statistical analysis in all subjects and for the diagnosis of hypertension in patients seen in endocrinology without known hypertension. The newly diagnosed hypertension was defined as a blood pressure of at least 140 mmHg systolic or 90 mmHg diastolic.

In patients seen in cardiology without previously diagnosed diabetes mellitus, OGTT was performed for the diagnosis of diabetes mellitus, which was defined as a plasma glucose concentration of at least 7.0 mmol/L at fasting or 11.1 mmol/L 2 h after ingestion of 75 g of glucose dissolved in water.

### Electrocardiogram (ECG) and urinary measurements

Standard 12-lead electrocardiogram (ECG) was recorded in all subjects. ECG left ventricular hypertrophy was defined according to the Cornell product index as (RaVL + SV3) × QRS duration > 244 mV·ms [8].

Urinary routine test was performed on fresh urine samples at the laboratory of each participating hospitals. Urinary albumin and creatinine excretions were measured using the immunochemical method in a core laboratory certified by the College of American Pathologists (https://www.cap.org). In the absence of apparent urological infections on urine samples, albuminuria was defined as a urinary albumin-to-creatinine ratio more than 30 mg/g. Albuminuria included both microalbuminuria (30–299 mg/g) and macroalbuminuria (≥ 300 mg/g).

### Other measurements

Anthropometric measurements included body weight, body height, and waist and hip circumferences. Body mass index was calculated as the body weight in kilograms divided by the body height in meters squared. Overweight was defined as a body mass index of 24.0 to 27.9 kg/m^2^, and obesity as a body mass index of 28 kg/m^2^ or greater. Central obesity was defined as a waist circumference of ≥ 90 cm for men and ≥ 85 cm for women.

Dyslipidemia was defined as a serum triglycerides concentration of 1.70 mmol/L or higher, a serum total cholesterol concentration of 5.18 mmol/L or higher, a serum low-density lipoprotein (LDL) cholesterol concentration of 3.37 mmol/L or higher, or a serum high-density lipoprotein (HDL) cholesterol concentration of 1.04 mmol/L or lower, or as the use of statin or other lipid lowering agents [9].

Ischaemic heart disease included myocardial infarction and angina. Ischaemic heart disease and stroke were self-reported.

### Statistical analysis

For database management and statistical analysis, we used SAS software (version 9.2, SAS Institute, Cary, NC, USA). Means and proportions were compared by the Student t-test and Fisher’s exact test, respectively. Continuous measurements with a skewed distribution were normalized by logarithmic transformation and represented by geometric mean and 95% confidence interval. Logistic regression analyses were performed to study the associations of interest.

## Results

### Demographics of the study population

Table 1 shows the demographics of 137 patients who were seen for hypertension in cardiology and had newly diagnosed diabetes mellitus and 155 patients who were seen for diabetes mellitus in endocrinology and had newly diagnosed hypertension. Hypertensive patients with newly diagnosed diabetes mellitus, compared to diabetic patients with newly diagnosed hypertension, had a significantly (*P *≤ 0.03) lower proportion of women (40.9% vs 54.8%) and were slightly older (2.4 ± 0.7 years).

**Table 1:** Characteristics of hypertensive patients with newly diagnosed diabetes mellitus and diabetic patients with newly diagnosed hypertension

Characteristic	Newly diagnosed diabetes mellitus (n = 137)	Newly diagnosed hypertension (n = 155)	*P* value
Men, n (%)	56 (40.9)	85 (54.8)	0.02
Age, years	60.0 ± 11.5	57.6 ± 10.8	0.06
Body mass index, kg/m^2^	26.3 ± 3.7	25.4 ± 3.5	0.03
Obesity, n (%)	43 (31.4)	31 (20.0)	0.03
Waist circumference, cm	92.1 ± 16.7	90.2 ± 10.2	0.26
Central obesity, n (%)	50 (52.1)	46 (47.9)	0.22
Current smoking, n (%)	22 (16.1)	32 (20.6)	0.31
Alcohol intake, n (%)	20 (14.6)	36 (23.2)	0.06
Systolic blood pressure, mmHg	144.2 ± 16.9	145.6 ± 15.1	0.47
Diastolic blood pressure, mmHg	84.2 ± 12.5	83.7 ± 10.7	0.73
Heart rate, beats/min	73.7 ± 12.7	78.1 ± 13.6	0.01
Plasma fasting glucose, mmol/l	6.78 (5.86–7.88)	8.00 (6.66–10.86)	< 0.0001
Plasma glycosylated haemoglobin A1c, %	6.71 ± 0.98	7.99 ± 1.97	< 0.0001
Serum triglycerides, mmol/l	1.52 (1.03–2.29)	1.34 (0.90–2.25)	0.73
Serum total cholesterol, mmol/l	4.98 ± 1.09	5.06 ± 1.18	0.57
Serum HDL cholesterol, mmol/l	1.30 ± 0.34	1.36 ± 0.42	0.25
Serum LDL cholesterol, mmol/l	3.01 ± 0.99	3.05 ± 0.96	0.73
Ischaemic heart disease, n (%)	23 (16.8)	10 (6.5)	0.01
Myocardial infarction, n (%)	4 (2.9)	1 (0.6)	0.14
Stroke, n (%)	9 (6.6)	5 (3.2)	0.18

Values are mean ± standard deviation, median (interquartile range) or number of subjects (%). For the definitions of obesity, central obesity, ischaemic heart disease, myocardial infarction and stroke, see “Methods”

*HDL* high-density lipoprotein, *LDL* low-density lipoprotein

### Clinical characteristics of newly diagnosed diabetes and newly diagnosed hypertension

Hypertensive patients with newly diagnosed diabetes mellitus, compared to diabetic patients with newly diagnosed hypertension, had significantly (*P *≤ 0.0001) lower plasma fasting glucose and HbA1c. However, they had similar systolic and diastolic blood pressure (*P *≥ 0.47). Among the other clinical characteristics, statistical significance (*P *≤ 0.03) was observed for body mass index and heart rate.

**Fig. 1:**
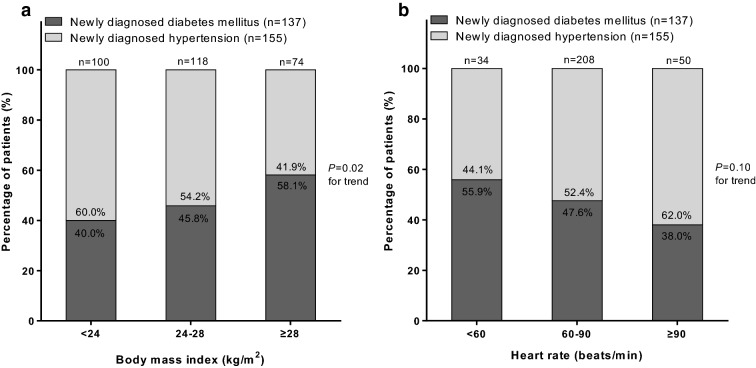
Percentage of hypertensive patients with newly diagnosed diabetes mellitus (solid) or diabetic patients with newly diagnosed hypertension (open) according to body mass index (**a**) and heart rate (**b**). The number and percentage of patients and *P* values for trend are given

Further categorical analyses (Fig. 1) according to body mass index (normal weight, overweight and obesity) and heart rate (< 60, 60–90, and ≥ 90 beats/min) showed that the percentage of hypertensive patients with newly diagnosed diabetes mellitus increased from 40.0% in normal weight subjects to 45.8% and to 58.1% in overweight and obese subjects, respectively (*P* for trend was 0.02) and decreased from 55.9% in patients with a heart rate below 60 beats/min to 47.6% and 38.0% in patients with a heart rate of 60–89 beats/min and ≥ 90 beat/min, respectively (*P* for trend was 0.10).

In sex and age-adjusted multiple logistic regression analyses, the odds ratio for newly diagnosed diabetes mellitus versus newly diagnosed hypertension was 1.27 for 3.0 kg/m^2^ greater body mass index (95% CI 1.03–1.56, *P *= 0.03) and 0.80 for 10 beats/min faster heart rate (95% CI 0.66–0.96, *P *= 0.02, Table 2).

**Table 2:** Odds ratio for newly diagnosed diabetes mellitus in hypertension (n = 137) versus newly diagnosed hypertension in diabetes (n = 155)

Variable	Odds ratio (95% confidence interval)	*P* value
Age (+ 10 years)	1.24 (0.99–1.56)	0.06
Male sex	1.75 (1.08–2.84)	0.02
Body mass index (+ 3 kg/m^2^)	1.27 (1.03–1.56)	0.03
Heart rate (+ 10 beats/min)	0.80 (0.66–0.96)	0.02

In a stepwise multiple logistic regression model, we forced age and sex and considered body mass index, waist-to-hip ratio, current smoking and alcohol intake, heart rate, serum triglycerides, and serum total and high-density cholesterol for entry and stay at a significance level of *P* ≤ 0.10

### Risk of albuminuria, left ventricular hypertrophy and ischaemic heart disease

**Fig. 2:**
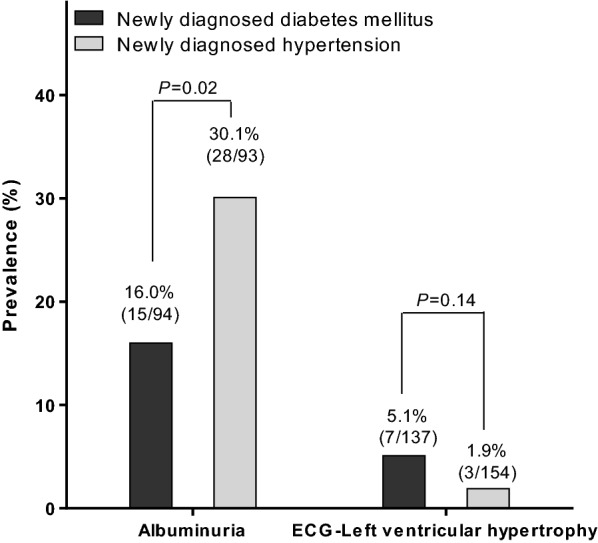
Prevalence of albuminuria and electrocardiographic (ECG)-left ventricular hypertrophy in hypertensive patients with newly diagnosed diabetes mellitus (solid bar, n = 137) or diabetic patients newly diagnosed hypertension (open bar, n = 155). The number and percentage of patients and *P* values are given

Hypertensive patients with newly diagnosed diabetes mellitus, compared to diabetic patients with newly diagnosed hypertension, had a significantly lower prevalence of albuminuria (16.0% vs. 30.1%, *P *= 0.02), higher prevalence of ischaemic heart disease (16.8% vs. 6.5%, *P *< 0.01) and slightly and non-significantly higher prevalence of ECG-left ventricular hypertrophy (5.1% vs. 1.9%, *P *= 0.14, Fig. 2).

After adjustment for sex, age, body height and body weight, current smoking and alcohol intake, mean arterial pressure, plasma glucose and serum triglycerides and total to HDL cholesterol ratio, the odds ratio in newly diagnosed diabetes mellitus versus newly diagnosed hypertension was 2.02 (95% CI 0.46–8.77, *P *= 0.35) for ECG left ventricular hypertrophy, 3.03 (95% CI 1.23–7.47, *P *= 0.02) for ischaemic heart disease and 0.53 (95% CI 0.29–0.96, *P *= 0.03) for albuminuria.

## Discussion

Our finding was that hypertensive patients with newly diagnosed diabetes mellitus had a greater body mass index and higher prevalence of left ventricular hypertrophy and ischaemic heart disease and a slower resting heart rate and lower prevalence of albuminuria than diabetic patients with newly diagnosed hypertension. Because the joint presence of these two clinical entities confers an even higher cardiovascular risk than either one alone, [2–4] our finding may have clinical implications in the prevention of this serious condition in those who already have either hypertension or diabetes mellitus alone.

Our observation on the risk of diabetes mellitus associated with a greater body mass index in patients with previously diagnosed hypertension is in keeping with the results of several previous prospective studies [10–14]. In 15,089 treated hypertensive patients attending the Glasgow Blood Pressure Clinic, body mass index was a major predictor of new onset diabetes mellitus (n = 1862) immediately after blood glucose level at baseline during 40 years of follow-up [10]. Similar results were observed in a post hoc analysis on the predictors of new-onset diabetes mellitus in 9995 hypertensive nondiabetic patients enrolled in the Valsartan Antihypertensive Long Term Use Evaluation (VALUE) trial [11]. Again, body mass index was the second important predictor of new-onset diabetes mellitus (n = 1298) during a mean follow-up of 4.2 years. Although there is strong evidence on the link between obesity and diabetes mellitus in hypertensive patients, obesity can also be a marker of the pathophysiological pathway shared between hypertension and diabetes mellitus, such as the insulin resistance [15].

Our observation on the risk of newly diagnosed hypertension associated with a faster heart rate in patients with an established diagnosis of diabetes mellitus is also in line with the concept that resting heart rate is a marker of cardiovascular risk, as demonstrated in numerous observational studies [16–19]. Resting heart rate is a measure of sympathovagal balance [19]. Faster resting heart rate could behave as an indicator of the impairment in sympathovagal function associated with diabetes mellitus. It is therefore not necessarily the resting heart rate itself that confers the risk of hypertension. It is the sympathovagal imbalance that renders the diabetic patients to develop high blood pressure. There is at least one study that has reported significant association between resting heart rate and the incidence of hypertension during a mean follow-up of 3.5 years [20]. In this study, diabetes mellitus was adjusted, but not analyzed separately from those non-diabetic patients.

Our explanation on the higher prevalence of left ventricular hypertrophy in hypertensive patients with a newly diagnosed diabetes mellitus and higher prevalence of albuminuria in diabetic patients with a newly diagnosed hypertension can be straight forward. Left ventricular hypertrophy is a major cardiac structural complication of hypertension [21]. Patients with a long-term established hypertension therefore have a higher risk of left ventricular hypertrophy than those with a newly diagnosed hypertension. Albuminuria is a complication of both diabetes mellitus and hypertension [22]. That explains why in the present study the prevalence of albuminuria was much higher than that of left ventricular hypertrophy. However, the prevalence of albuminuria is high in the presence of diabetes mellitus only than in the presence of hypertension only [23, 24]. That explains why in the present study, diabetes mellitus with a newly diagnosed hypertension had a higher risk of albuminuria.

Our study should be interpreted within the context of its limitations. First, our study had a cross-section design and does not allow any causal inference. Although our patients had newly diagnosed hypertension or diabetes, our study was still different from a prospective observational one. Second, our study had a relatively small sample size with few measurements of biological markers. Third, albuminuria was evaluated on a single spot urine sampling. However, albuminuria and creatinine were measured in a core laboratory. A stringent quality assurance programme was implemented, including the exclusion of patients with suspected urinary tract infections. Fourth, we did not collect information on the frequency of patients’ clinic visit. Hypertensive and diabetic patients in China are usually followed up on a monthly basis for drug prescriptions. However, it is still possible that the time interval varied between patients, which might influence the between-group comparisons.

## Conclusion

In conclusion, earlier or later onset of hypertension than diabetes mellitus may have different risk predictors and organ damages. Our finding should be tested in prospective intervention studies on the possible pathophysiological pathways behind obesity and tachycardia.

## Supplementary information

**Additional file 1: Appendix 1.** Participating hospitals of the China ATTEND Registry.

## Data Availability

Qualified researchers may request access to patient level data and related study documents including the clinical study report, study protocol with any amendments, blank case report forms, statistical analysis plan, and dataset specifications. Patient level data will be anonymized and study documents will be redacted to protect the privacy of our study participants. Further details on Sanofi’s data sharing criteria, eligible studies, and process for requesting access can be found at: https://www.clinicalstudydatarequest.com/.

## Abbreviations

HbA1c: glycosylated haemoglobin A1
OGTT: oral glucose tolerance test
ECG: electrocardiogram
LDL: low-density lipoprotein
HDL: high-density lipoprotein
BMI: body mass index
