# Supplementary material for: Comparison between newly diagnosed hypertension in diabetes and newly diagnosed diabetes in hypertension
Source: Diabetol Metab Syndr. 2019 Aug 23;11:69. doi: 10.1186/s13098-019-0465-3 (PMC6708242; doi:10.1186/s13098-019-0465-3)
Supplement: Supplementary file 1 — Additional file 1: Appendix 1. Participating hospitals of the China ATTEND Registry. [file 13098_2019_465_MOESM1_ESM.docx]

**Appendix 1.** **Participating hospitals of the China ATTEND Registry**

The participating hospitals were listed in the alphabetical order of province and hospital, with departments, principal investigators and the number of enrolled patients in the parentheses.

*Beijing:* Anzhen Hospital (Cardiology, Chang-Sheng Ma, n=70; Endocrinology, Yi Zhao, n=60), Chaoyang Hospital (Endocrinology, Yuan Xu n=63), Tongren Hospital (Endocrinology, Jin-Kui Yang, n=60) and Xuanwu Hospital (Cardiology, Dong Xu, n=50; Endocrinology, Li Wang, n=53), Capital Medical University; Peking Union Medical College Hospital (Cardiology, Quan Fang, n=90; Endocrinology, Xiao-Ping Xing, n=54); First Hospital (Cardiology, Jie Jiang, n=75; Endocrinology, Xiao-Hui Guo, n=55) and People’s Hospital (Cardiology, Da-Yi Hu, n=70; Endocrinology, Li-Nong Ji, n=60), Peking University; *Fujian:* Fujian Medical University Union Hospital (Cardiology, Liang-Long Chen, n=76; Endocrinology, Li-Bin Liu, n=60); *Guangdong:* Guangdong Province People’s Hospital (Cardiology, Hua Yao, n=76), Guangzhou; *Hunan:* The Third Xiangya Hospital (Cardiology, Kan Yang, n=71; Endocrinology, Zhao-Hui Mo, n=60) and Xiangya Hospital (Cardiology, Tian-Lun Yang, n=71; Endocrinology, Min-Xiang Lei, n=51), Central South University, Changsha; *Jiangsu:* Jiangsu Province People’s Hospital (Cardiology, Ke-Jiang Cao, n=50; Endocrinology, Tao Yang, n=60), Nanjing; General Hospital of Nanjing Military Command (Cardiology, Jian-Bin Gong, n=87; Endocrinology, Jian Wang, n=60), Nanjing; Wuxi People’s Hospital (Cardiology, Zhen-Yu Yang, n=70; Endocrinology, Rui-Fang Bu, n=70); *Jiangxi:* The First Affiliated Hospital of Nanchang University (Cardiology, Meng-Hong Wang, n=70; Endocrinology, Jian-Ying Liu, n=51); *Jilin:* The First Affiliated Hospital (Cardiology, Yang Zheng, n=70; Endocrinology, Gui-Xia Wang, n=70) and The Second Affiliated Hospital (Endocrinology, Yu Liu, n=60), Jilin University, Changchun; *Liaoning:* The People’s Hospital of Liaoning Province (Cardiology, Zhan-Quan Li, n=60), Shenyang; *Shanghai:* Renji Hospital (Endocrinology, Wei Liu, n=55), Ruijin Hospital (Cardiology, Ji-Guang Wang, n=71) and Shanghai First People’s Hospital (Cardiology, Shao-Wen Liu, n=77), Shanghai Jiaotong University School of Medicine; *Sichuan*: Sichuan Province People’s Hospital (Cardiology, Jian-Hong Tao, n=3; Endocrinology, Peng-Qiu Li, n=60), Chengdu; West China Hospital (Cardiology, Xiao-Ping Chen, n=39; Endocrinology, Hao-Ming Tian, n=58), Sichuan University, Chengdu; *Zhejiang:* The First Affiliated Hospital (Endocrinology, Cheng-Jiang Li, n=60) and The Second Affiliated Hospital (Cardiology, Jian-An Wang, n=84), Zhejiang University, Hangzhou.
